# Are lung cysts in renal cell cancer (RCC) patients an indication for *FLCN* mutation analysis?

**DOI:** 10.1007/s10689-015-9853-5

**Published:** 2015-11-24

**Authors:** Paul C. Johannesma, Arjan C. Houweling, Fred H. Menko, I. van de Beek, Rinze Reinhard, Johan J. P. Gille, JanHein T. M. van Waesberghe, Erik Thunnissen, Theo M. Starink, Pieter E. Postmus, R. Jeroen A. van Moorselaar

**Affiliations:** Department of Pulmonary Diseases, VU University Medical Center, PO Box 7057, 1007 MB Amsterdam, The Netherlands; Department of Clinical Genetics, VU University Medical Center, Amsterdam, The Netherlands; Family Cancer Clinic, The Netherlands Cancer Institute, Amsterdam, The Netherlands; Department of Radiology, VU University Medical Center, Amsterdam, The Netherlands; Department of Pathology, VU University Medical Center, Amsterdam, The Netherlands; Department of Dermatology, Leiden University Medical Center, Leiden, The Netherlands; Department of Molecular and Clinical Cancer Medicine, Clatterbridge Cancer Centre, Liverpool Heart and Chest Hospital, University of Liverpool, Liverpool, UK; Department of Urology, VU University Medical Center, Amsterdam, The Netherlands

**Keywords:** Birt–Hogg–Dubé syndrome, BHD, Folliculin, Pneumothorax, Renal cell cancer, RCC

## Abstract

Renal cell cancer (RCC) represents 2–3 % of all cancers and is the most lethal of the urologic malignancies, in a minority of cases caused by a genetic predisposition. Birt–Hogg–Dubé syndrome (BHD) is one of the hereditary renal cancer syndromes. As the histological subtype and clinical presentation in BHD are highly variable, this syndrome is easily missed. Lung cysts—mainly under the main carina—are reported to be present in over 90 % of all BHD patients and might be an important clue in differentiating between sporadic RCC and BHD associated RCC. We conducted a retrospective study among patients diagnosed with sporadic RCC, wherein we retrospectively scored for the presence of lung cysts on thoracic CT. We performed *FLCN* mutation analysis in 8 RCC patients with at least one lung cysts under the carina. No mutations were identified. We compared the radiological findings in the *FLCN* negative patients to those in 4 known BHD patients and found multiple basal lung cysts were present significantly more frequent in *FLCN* mutation carriers and may be an indication for BHD syndrome in apparent sporadic RCC patients.

## Introduction

Renal cell carcinoma (RCC) represents 2–3 % of all cancers and is the most lethal of the urologic malignancies. There has been an annual increase about 2 % in the incidence, with 88,400 new cases of RCC worldwide in 2008 [1]. The incidence is approximately 5.8 per 100.000 and the mortality is approximately 1.4 per 100.000 [2]. Currently over 50 % of RCC are detected incidentally, as only a minority (6–10 %) of patients present with the classic triad; flank pain, gross haematuria and palpable abdominal mass [3]. These data underline the value of pre symptomatic identification and screening of patients with an increased risk for RCC. A genetic predisposition for renal cancer is currently estimated to be present in 3–5 % of RCC patients, often showing recognisable features in addition to the increased risk for renal cancer [4]. A probably underdiagnosed autosomal dominant cancer disorder is the Birt–Hogg–Dubé syndrome (BHD), clinically characterized by skin fibrofolliculomas, lung cysts in >90 % of cases, (recurrent) spontaneous pneumothorax (SP) and an increased lifetime risk for RCC between 16 and 35 %. The gene associated with BHD encodes the protein folliculin (*FLCN*) which acts as a tumour suppressor and interacts with mTOR and AMPK signalling pathways [5]. The incidence is estimated about 1 in 200.000 people [6]. So far, two large studies described the phenotypes of a large cohort of families with BHD. The first study by Toro and colleagues showed 24 cases with a history of RCC. Of them 22/24 (91.7 %) had multiple lung cysts on thoracic CT. In the remaining two patients, no thoracic CT scan was available. The second study performed in our University centre, we found 17 cases of RCC among a total of 115 *FLCN* mutation carriers. A thoracic CT was available in 13/17 (76.4 %) which showed cysts in one or both lungs in all cases. In the other four cases no thoracic CT was available [7, 8].

Therefore we hypothesized, that cysts under the main carina in patients with diagnosed with “sporadic” RCC might be an important diagnostic clue in unmasking Birt–Hogg–Dubé syndrome. We performed a pilot study to evaluate this hypothesis.

## Materials and methods

**Fig. 1:**
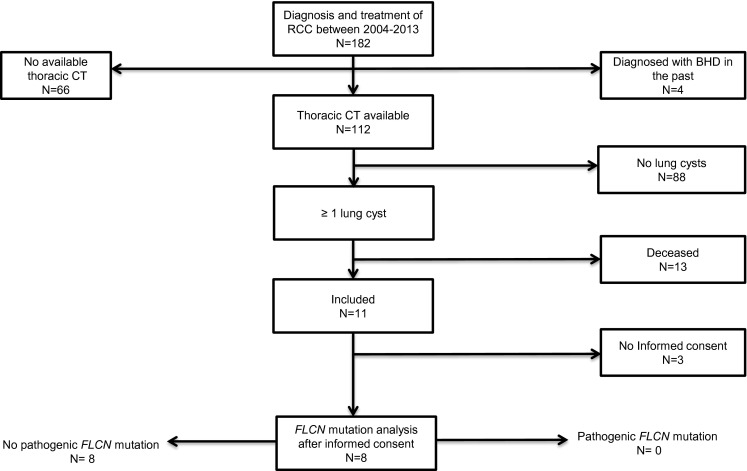
Study in-/exclusion flowchart

We retrospectively collected data on all patients (n = 182), who had been diagnosed and treated for RCC in the years 2003–2013. Patients diagnosed with RCC in our center were included when they were over the age of 18 years at the time of diagnosis. Exclusion criteria were metastasis in the kidney, no available thoracic CT or patients already known with a proven pathogenic *FLCN* mutation. Furthermore, deceased patients were excluded (Fig. 1). All thoracic CT’s, made in the period 2003–2013 were collected and scored by one radiologist for the presence of one or more lung cysts, below the level of the carina. Furthermore we collected the clinical data on familial occurrence on SP, RCC and the history of SP. Clinical information was not available to the radiologist at the time of scoring. We compared the radiological data to that of 4 *FLCN* mutation carriers diagnosed with RCC and tested for potential significant differences in the number and size of lung cysts.

## Results

### Patient data

#### Proven *FLCN* mutation carriers (N = 4)

In our BHD cohort of 250 *FLCN* mutation carriers, 4 patients were diagnosed with BHD after the diagnosis of RCC and this enabled screening of their relatives. All 4 index patients had at diagnosis one or more symptoms of the classic triad; flank pain, gross haematuria and/or a palpable abdominal mass at the time of diagnosis. All 4 patients had one or more RCC’s on the abdominal CT. The mean age at time of diagnosis was 45.4 years of age (31–63 years).One patient had bilateral RCC and one patient had two renal tumours in one kidney. Histopathology showed RCC with clear cell and chromophobe elements in three patients and chromophobe elements only in one patient. A thoracic CT was performed in all four patients that showed between 1 and 51 lung cysts in the basal parts of the lung. Recurrent episodes of pneumothorax (3 episodes) occurred in one patient. Three patients had multiple fibrofolliculomas in the face and upper neck. The family history for SP was positive in three patients and was positive in two patients for RCC in two patients (Table 1).

**Table 1:** Characteristics of 4 RCC patients with pathogenic *FLCN* mutation and 8 RCC patients without a pathogenic *FLCN* mutation

Patient	RCC				Fam. predisposition		SP in medical history (N)	Lung cysts (N)	*Fibrofolliculomas*	*FLCN* mutation
	Histopathology	Treatment	Diagnosis (age)	Location	SP	RCC				
1	Chromophobe	Partial	63	Left + right	0	0	0	1	Yes	c.774_775delGTinsCAC
2	Chromophobe	Partial	31	Left (N = 2)	1	0	3	24	Yes	c.499C>T
3	Chromophobe	Partial	56	Left	1	1	1	51	No	c.610_611delGCinsTA
4	Chromophobe	RFA	41	Left	1	1	1	16	Yes	c.1552delC
5	Clearcell	Partial	63	Right	0	0	0	1	No	No
6	Chromophobe	Total	73	Right	0	0	0	2	No	No
7	Clearcell	Total	60	Right	0	0	0	2	No	No
8	Clearcell	Partial	53	Left	0	0	0	1	No	No
9	Sarcomatoid	Total	62	Left	0	0	0	1	No	No
10	Clearcell	Partial	73	Left	0	0	0	1	No	No
11	Clearcell	Total	69	Right	0	0	0	2	No	No
12	Clearcell	Partial	66	Left	0	0	3	5	No	No

#### *FLCN* negative RCC patients (N = 8)

For the evaluation for the potential presence of BHD in a “sporadic” RCC cohort, we included 182 patients with sporadic RCC in the medical history. Between 2003 and 2013 a total of 112 patients underwent one or more thoracic CT’s, in the remaining 70 patients only a chest X-ray was performed. Eleven patients met our inclusion criteria. Of these eight patients gave informed consent for a one time visit at our outpatient clinic (Fig. 1). The mean age was 64.9 years (53–73 years) at time of diagnosis. On thoracic CT all eight patients had at least one cyst in the basal parts of the lung. Four patients had one cyst in the basal parts of the lung, three patients had two cysts in the basal parts of the lung and one patient had 5 cysts in the basal parts of the lung respectively. None of the patients had bilateral or multifocal RCC. The histopathology was clear cell in six patients, chromophobe in one patient and sarcomatoid in one patient. The patient with 5 lung cysts had a history of three episodes of SP; the other 7 patients had never experienced a SP. The familial history for SP and RCC was negative in all eight patients. *FLCN* mutation analysis was performed; in none of the eight patients a pathogenic *FLCN* mutation was found (Table 1).

## Discussion

To date, ten hereditary renal cancer syndromes have been defined, accounting for 3–5 % of all RCCs [4]. Based on the reported increased presence of multiple lung cysts under the carina in BHD patients we performed *FLCN* mutation analysis in a pilot study setting among 8 patients with a history of RCC and one or more lung cysts in the basal parts of the lung on thoracic CT. Although the history of smoking was not available in all patients, it was possible to distinguish between smoking related bullae in the apical parts of the lung and lung cysts on thoracic CT, as described by Fabre et al. [9] We found no pathogenic *FLCN* mutations in this group. Although no pathogenic *FLCN* mutation is found in this group, BHD is not excluded as only in 81–84 % of clinical BHD cases a pathogenic germline *FLCN* mutation is found [10]. We compared the analysed the radiological findings to those in 4 proven *FLCN* mutation carriers with renal cancer. We found that multiple basal lung cysts were present significantly more frequent in *FLCN* mutation carriers and may be an indication for further evaluation of BHD syndrome in apparently sporadic RCC patients. However, since solitary cysts were found in both groups, the absence of multiple cysts does not appear to be a specific marker for the absence of BHD, as one BHD patient had only one cyst on thoracic CT. We therefore advise that in all RCC patients at least a concise family history is taken for the presence of RCC or SP and the skin is evaluated for the presence of fibrofolliculomas. In the presence of a positive family history (SP or RCC) or multiple basal lung cysts further investigation of BHD is indicated (e.g. by dermatological evaluation or by DNA testing). The difficulty in unmasking BHD patients in apparently sporadic RCC patients is illustrated by the negative family history for pneumothorax and RCC in the two *FLCN* mutation carriers. Based on our results, we conclude that lung cysts in RCC patients can be an indicator for underlying Birt–Hogg–Dubé syndrome, although the absence of presence of solitary lung cysts doesn’t equate to the diagnosis of BHD. Further evaluation in larger RCC cohorts is required to confirm our findings.

## Abbreviations

BHD: Birt–Hogg–Dubé syndrome
CT: Computed tomography
FLCN: Folliculin
RCC: Renal cell cancer
SP: Spontaneous pneumothorax
